# Effects of foot–ground friction and age-related gait changes on falls during walking: a computational study using a neuromusculoskeletal model

**DOI:** 10.1038/s41598-024-81361-7

**Published:** 2024-11-28

**Authors:** Naoto Izumi, Takashi Yoshida, Toshiaki Nishi, Kei Masani, Takeshi Yamaguchi

**Affiliations:** 1 Graduate School of Engineering, Tohoku University, 6-6-01 Aramaki-Aza-Aoba, Aoba-ku, Sendai, 980-8579 Miyagi Japan; 2 Institute of Biomedical Engineering, University of Toronto, Toronto, ON Canada; 3 Lyndhurst Centre, KITE Research Institute–University Health Network, Toronto, ON Canada; 4 Graduate School of Biomedical Engineering, Tohoku University, 6-6-01 Aramaki-Aza-Aoba, Aoba-ku, Sendai, 980-8579 Miyagi Japan

**Keywords:** Neuromusculoskeletal bipedal gait model, Slip, Trip, Fall, Coefficient of friction, Aging, Biomedical engineering, Ageing

## Abstract

We used a neuromusculoskeletal model of bipedal walking to examine the effects of foot–ground friction conditions and gait patterns on slip- and trip-induced falls. We developed three two-dimensional neuro-musculoskeletal models in a self-organized manner representing young adults, elderly non-fallers, and elderly fallers. We simulated walking under different foot-ground friction conditions. The static friction coefficient between the foot and the ground was varied from 0.05 to 2.0. Under low friction conditions, the three gait models demonstrated slip-induced falls. The elderly faller model experienced the most slip. This is because the RCOF was higher in the elderly faller model due to its short stride length but much smaller foot clearance. Under high friction conditions, only the elderly faller model demonstrated trip-induced falls. Based on the analysis using the margin of stability, the forward postural stability of the model gradually decreased under high-friction conditions, with the toe of the swing foot contacting the ground and subsequently falling forward. These results imply that there is an optimal coefficient of friction for the ground to prevent slip- and trip-induced falls by people with less stable gaits, which may provide new insights into the design of shoes and floor surfaces for the elderly.

**Supplementary Information:**

The online version contains supplementary material available at 10.1038/s41598-024-81361-7.

## Introduction

One factor that threatens the long-term health of the elderly is falling accidents^1^, which are mainly caused by slips and trips^2^. The aging process negatively affects the gait by reducing the gait speed, step length^3,4^. Elderly fallers, defined as having a history of at least one fall in the previous year, exhibit increased variability in these gait parameters^5–7^. It has been reported that elderly fallers have a smaller minimum toe clearance, which is a biomechanically defined event during the mid-swing phase of the gait cycle, than healthy young adult^8^. It is also pointed out that the elderly are more likely to trip when the median values of minimum foot clearance are low and when minimum foot clearance variability is high^9^.

A shorter step length decreases the required coefficient of friction (RCOF), which is the maximum ratio of horizontal to vertical ground reaction forces during the stance phase^10,11^. A reduced foot clearance increases the horizontal heel velocity at heel contact, resulting in an increase in RCOF^12^. Therefore, there is a trade-off between decreased stride length and decreased foot clearance with respect to slip risk, and a gait with a greater stride length/foot clearance ratio is thought to be associated with a higher risk of slip. Studies^13,14^ have shown that a higher gait speed decreases the risk of slip-induced falls in young adults. Therefore, a low gait speed seen in the elderly may also increase the risk of a slip-induced fall.

Meanwhile, the reduced foot clearance and large variability in gait parameters of elderly fallers may also increase accidental and unexpected contact between the ground and toe during the mid-swing phase, which can increase the risk of trip-induced falls. In addition to steps and other obstacles, Menant et al.^15^ noted that excessive slip-resistance could lead to tripping in older people, even on a floor with level surface, although there are no experimental validation results for this observation. They also suggested that too much friction between the shoe and the walking surface could undermine stability among older people^15^. Other literature also points out the possibility of trip-induced falls when foot-floor friction is excessive in the absence of tripping hazard such as objectives and steps on the floor^16,17^. Yamaguchi et al.^18^ simulated the gait of elderly fallers on a level floor surface and reported that the external forward moment about the body center of mass (CoM) increases with an increasing foot–ground coefficient of friction, causing tripping risk. In addition, case studies by Nemire et al.^19^ indicated the possibility of the occurrence of trip-induced falls due to high friction on level floor with no tripping hazard in the elderly. They pointed out that the primary conditions for these tripping events are wearing shoes with high-traction soles while walking on surfaces that provide a high level of slip resistance.

Several studies have investigated the relationship between slip occurrence or slip-induced falls and the coefficient of friction between the shoe sole and the floor. Hanson et al.^20^ investigated the relationship between shoe–floor coefficient of friction and slip-induced falls and estimated the probability of a fall event from the difference between the measured coefficient of friction and the RCOF. Iraqi et al.^21^ also developed a probability model for slip prediction using the measured coefficient of friction and RCOF. Fong et al.^22^ determined the limit of the coefficient of friction which human starts to walk carefully to adapt to slippery surface to be 0.41. Mahboobin et al.^23^ used computer simulations to investigate changes in walking behavior due to a decrease in the coefficient of friction between the shoe sole and the floor. They found that when slip occurred under low-friction conditions, the subject’s lower limb movements were different from those when the subject was walking on a dry surface with high friction. These studies mainly focused on slips and slip-induced falls under low-friction conditions, and their results were based on walking experiments that involved healthy, young adult participants or computer simulations that modeled the gait of a healthy young adult. Therefore, no studies focused on trips and/or trip-induced falls due to high foot–ground friction, and none investigated the relationship between foot–ground friction and age-related changes in gait characteristics.

In this study, we carried out computational simulations using a neuromusculoskeletal model of bipedal walking^24^ to investigate the relation between foot–ground friction and the risk of slip- or trip-induced falls. We hypothesized that a gait observed among elderly fallers is more likely to result in slip-induced falls under low foot–ground friction conditions. Additionally, we hypothesized that large foot–ground friction is a critical factor for trip-induced falls, especially among elderly fallers.

## Results

**Fig. 1:**
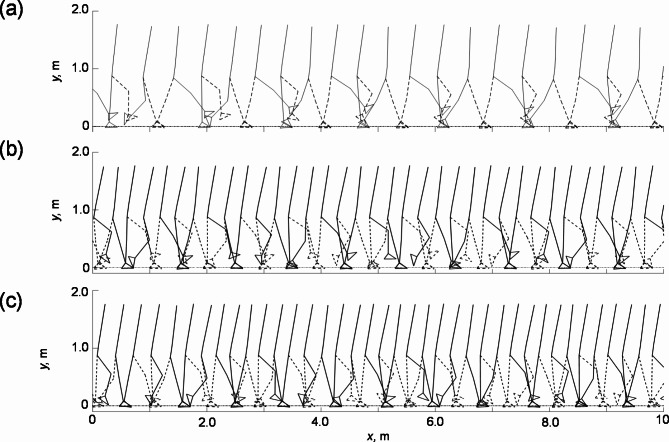
Stick diagrams of gaits obtained by computer simulations over a distance of 10 m without the static coefficient of friction (*µ*_s_) being set: (a) the young adult model, (b) elderly non-faller model, and (c) elderly faller model.

The results of each gait model were plotted as stick diagrams (Fig. 1). Table 1 lists the mean of the stride length, walking speed, minimum foot clearance, and maximum foot clearance, as well as their coefficient of variation (CV). Compared to the young adult model, the elderly non-faller and elderly faller models reduced their stride lengths by 35% and 40%, respectively, their gait speeds by 42% and 47%, respectively, their maximum foot clearances by 22% and 58%, respectively, and their minimum foot clearances by 13% and 30%, respectively. Meanwhile, they increased the CVs of the minimum foot clearance from 6.73 to 23.1% and 45.6%, respectively. The differences between the three gait models were qualitatively consistent with the differences in gait among young adults, elderly non-fallers, and elderly fallers as reported in the previous literature^25–28^.

**Table 1:** Mean and coefficient of variation (CV) of the stride lengths, walking speeds, and maximum and minimum foot clearances of the three gait models.

Variables		Young adult model	Elderly non-faller model	Elderly faller model
Stride length	Mean, m	1.47	0.95	0.88
	CV, %	1.34	0.99	7.29
Walking speed	Mean, m/s	1.29	0.75	0.69
	CV, %	1.68	0.52	10.8
Maximum foot clearance	Mean, m	0.36	0.28	0.15
	CV, %	2.2	3.6	26.0
Minimum foot clearance	Mean, mm	20.8	18.1	14.6
	CV, %	6.73	23.1	45.9

**Fig. 2:**
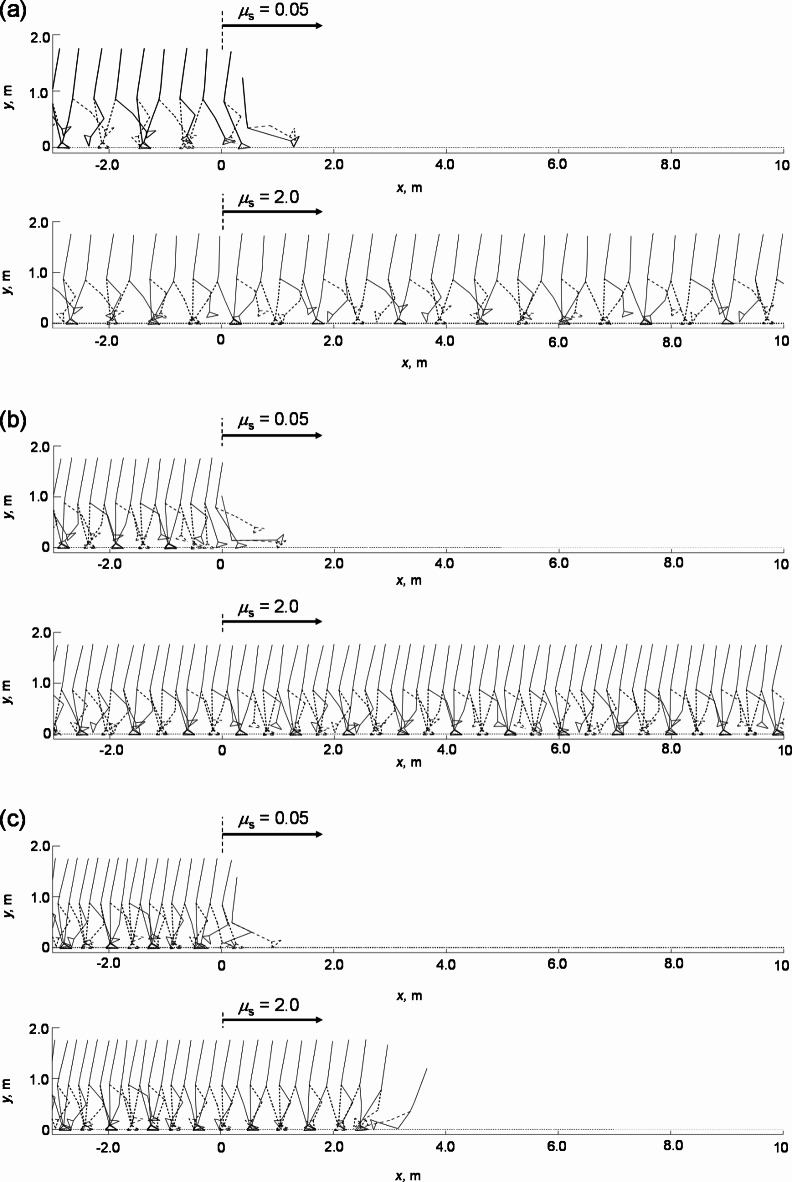
Stick diagrams of gaits obtained by computer simulations at *µ*_s_ = 0.05 and *µ*_s_ = 2.0: (a) the young adult model, (b) elderly non-faller model, and (c) elderly faller model.

Figure 2 shows the stick diagrams obtained with the three gait models under low- (*µ*_s_ = 0.05) and high-friction conditions (*µ*_s_ = 2.0) between the feet and ground. For the young adult model (Fig. 2a) and elderly non-faller model (Fig. 2b), a large forward slip occurred the first step after *µ*_s_ changed to 0.05, which resulted in a backward slip-induced fall, while no fall was observed when *µ*_s_ was 2.0.Thus, the elderly non-faller model showed the same trend as the young adult model. For the elderly faller model (Fig. 2c), a large forward slip occurred the first step after *µ*_s_ changed to 0.05 resulting in a backward fall, similar to the other two models. Additionally, the swing foot of the elderly faller model contacted the ground the fourth step after *µ*_s_ changed to 2.0. The body then leaned forward and subsequently fell forward.

**Fig. 3:**
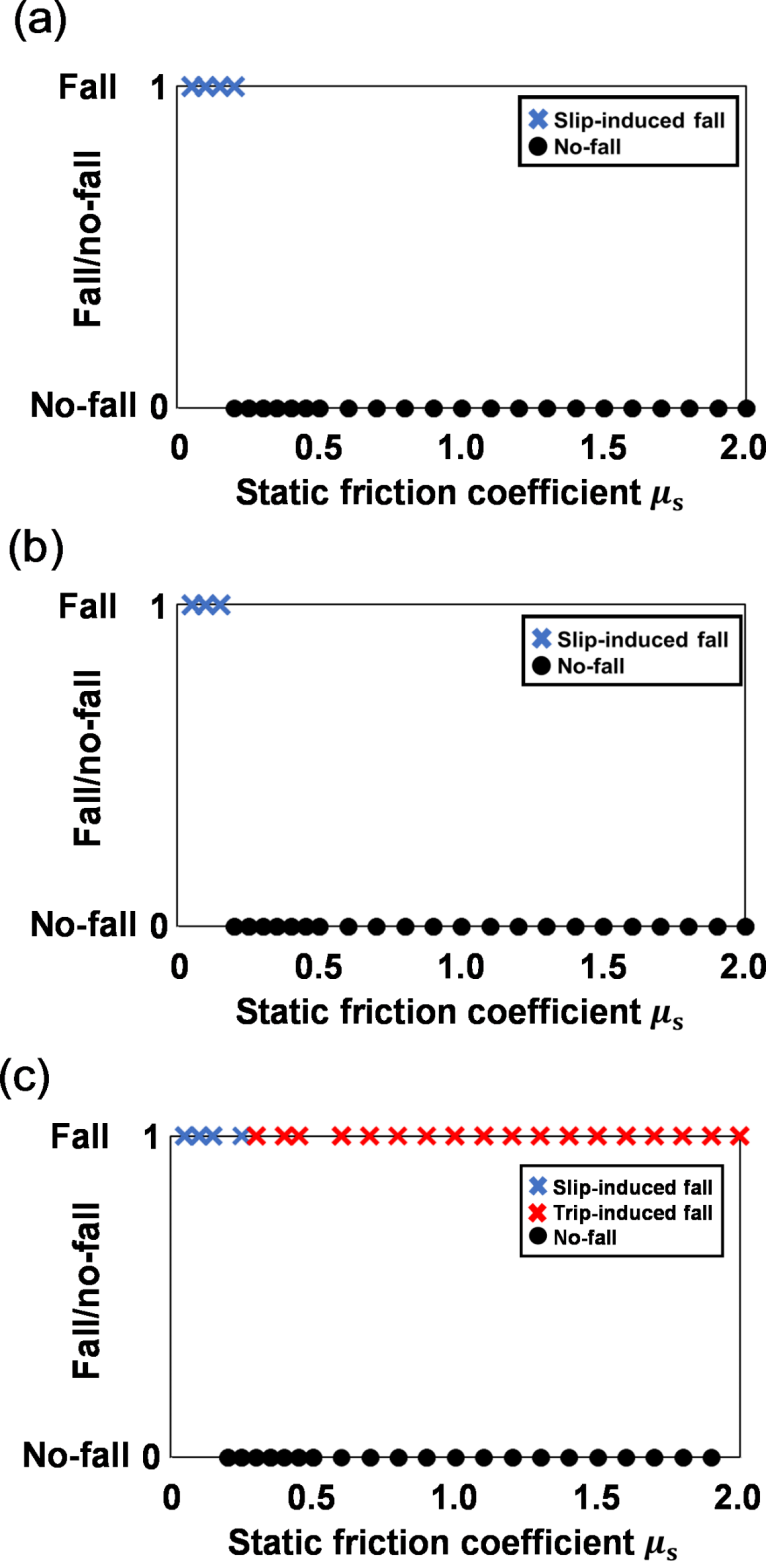
Relation between *µ*_s_ and falls: (a) the young adult model, (b) elderly non-faller model, and (c) elderly faller model. The black dots represent no-falls (0), and the crosses represent falls (1). Blue falls are slip-induced, and red falls are trip-induced. Each graph includes all simulation results and thus has 125 plots.

Figure 3 shows the relation between *µ*_s_ and the fall occurrence of the three gait models. At low *µ*_s_, all three models experienced slip-induced falls. The young adult model (Fig. 3a) experienced slip-induced falls when *µ*_s_ ≤ 0.20 and no falls when *µ*_s_ ≥ 0.25. The elderly non-faller model (Fig. 3b) experienced slip-induced falls when *µ*_s_ ≤ 0.15 and no falls when *µ*_s_ ≥ 0.20. These models experienced low-traction between the foot and ground even at high *µ*_s_, so no trip-induced falls occurred (see Fig. A1). The elderly faller model (Fig. 3c) experienced slip-induced falls when *µ*_s_ ≤ 0.25 and trip-induced falls when *µ*_s_ ≥ 0.30, which differed from the trends of the other two models.

**Fig. 4:**
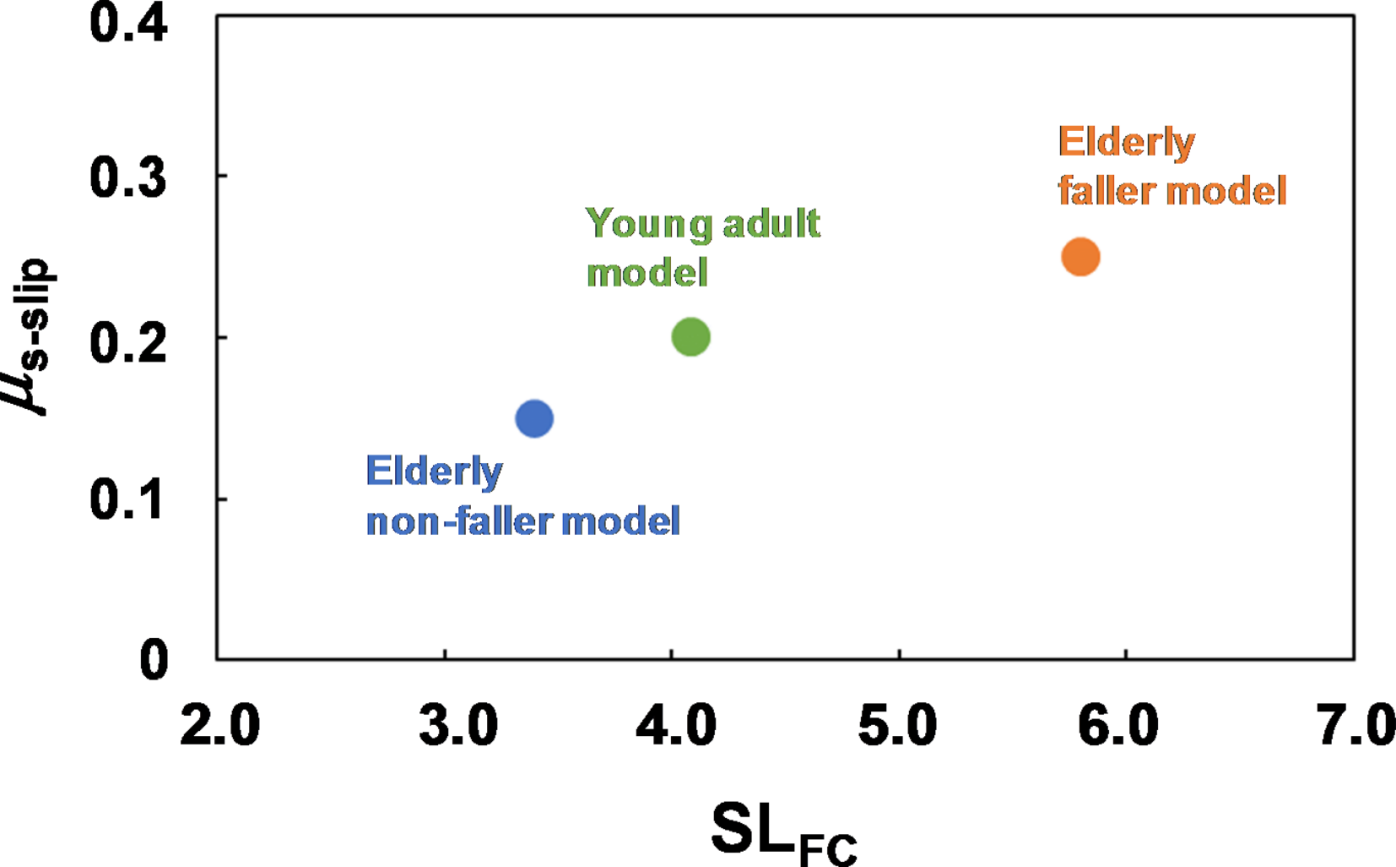
Relation between the upper limit of *µ*_s_ for slip-induced falls (*µ*_s−slip_) and the step length with respect to foot clearance (SL_fc_).

Figure 4 shows the relationship between the ratio of stride length to foot clearance SL_FC_ and the upper limit of *µ*_s_ for a slip-induced fall *µ*_s−slip_ for three gait models. As shown in Fig. 4, *µ*_s−slip_ increases with increasing SL_FC_, and the both *µ*_s−slip_ and SL_FC_ for the elderly faller model was largest among the gait models.

**Fig. 5:**
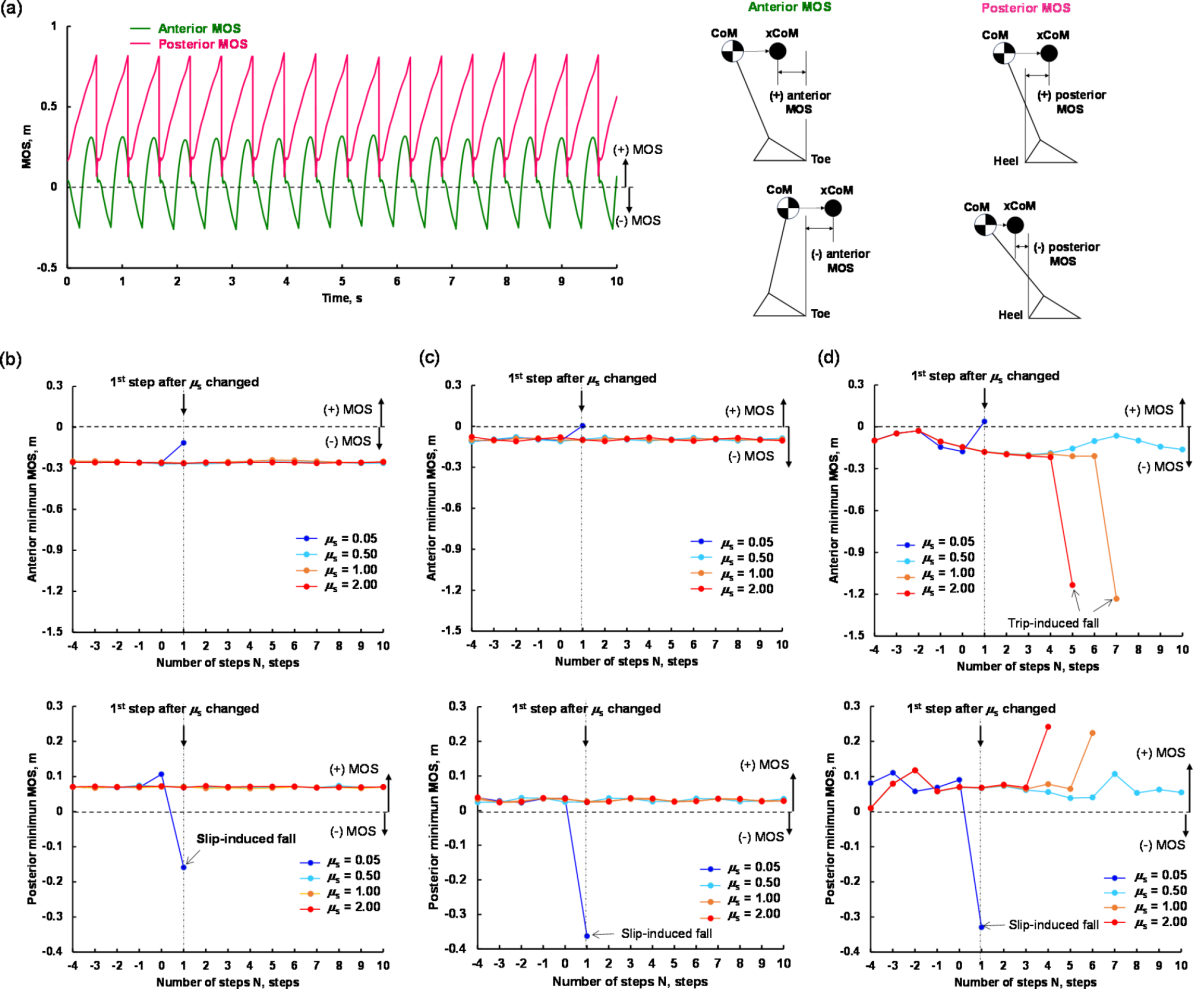
Relation between the minimum margin of stability(MOS) value in the anterior and posterior directions during a single step and the number of steps after a change in the ground friction condition: (a) Example of time variation of the anterior and posterior MOS for a stable gait in the young adult model and the definition of negative and positive MOS; (b) young adult model, (c) elderly non-faller model; and (d) elderly faller model.

Figure 5 presents the plots of the minimum anterior and posterior margin of stability (MOS) values of the three gait models for each step under four different *µ*_s_ conditions (*µ*_s_ = 0.05, 0.5, 1.0, and 2.0) after the *µ*_s_ condition changed (i.e., *x* > 0 m in Fig. 2). As shown in Fig. 5b–d, when a slip induced fall occurs at *µ*_s_ = 0.05, the posterior minimum MOS changes from positive to negative due to a forward slip, increasing backward instability. The minimum posterior MOS after the model steps on a low friction floor is lower for the young adult gait model than for the other elderly gait models. On the other hand, as shown in Fig. 5d, when a trip-induced forward fall occurs under the high-friction conditions of *µ*_s_ = 1.0 and *µ*_s_ = 2.0, the anterior minimum MOS decreases gradually and then drops sharply, resulting in greater forward instability and a fall. The number of steps, which caused a spike in forward instability, taken after the change in frictional condition is shorter at *µ*_s_ = 2.0 (fifth step) than at *µ*_s_ = 1.0 (seventh step).

## Discussion

### Analysis of results under low-friction conditions

Our first hypothesis was that a gait with a small foot clearance such as that observed among elderly fallers might be more prone to slips. As expected, the elderly faller model had more slip-induced falls than the other two gait models. The *µ*_s−slip_ was highest for the elderly faller model (*µ*_s−slip_ = 0.25), followed by the young adult model (*µ*_s−slip_ = 0.20) and elderly non-faller model (*µ*_s−slip_ = 0.15). The fewer slip-induced falls for the elderly non-faller model compared to the young adult model at low *µ*_s_ can be attributed to a shorter stride length (Table 1). A smaller stride length decreases the RCOF^29^, which results in a gait that is more resistant to slips. Experimental studies confirm that elderly non-fallers walk with a small stride length and reduced RCOF, which increases their resistance to slips^30^ and supports our computer simulation results. However, the elderly faller model had the smallest stride length but experienced the most slip-induced falls. The elderly faller model exhibited the smallest foot clearances among the three gait models. A gait with reduced foot clearance increases the horizontal velocity of the heel at contact, which results in higher RCOF^12^. Although the elderly faller model had the smallest stride length and foot clearance among the three gait models, it had the largest RCOF due to the largest stride length/foot clearance as shown in Fig. 4. Consequently, lower foot clearance in the elderly faller model is assumed to significantly contribute to the elevated risk of slip-induced falls. Another reason that the elderly faller model was more prone to falls under the low-friction condition might have been the low gait speed. The higher the gait speed, or the more forward the body CoM, the greater the stability against backward loss of balance^13,14^. This is supported by the fact that the minimum posterior MOS of the young adult model at *µ*_s_ = 0.05 is larger than that of the elderly non-faller model and the elderly faller model, as shown in Fig. 5b–d.

### Analysis of results under high-friction conditions

Our second hypothesis was that the foot–ground friction is a critical factor for trip- and slip-induced falls. The simulation results showed that only the elderly faller model experienced trip-induced forward falls (Fig. 3c). For this model, the toe tip in the mid-swing phase contacted the ground at high *µ*_s_ (Fig. 2c), which caused the trip-induced falls.

Interestingly, when the trip-induced fall occurred under high friction condition as shown in Fig. 2c, the swing foot did not contact the ground the first step after *µ*_s_ changed but the fifth step. This is also true for the change in minimum anterior MOS versus the number of steps after the *µ*_s_ changed (Fig. 5d). That is, after the friction condition changes, the instability increases sharply at the fifth step. In the elderly faller model, the MOS decreases gradually before dropping sharply for *µ*_s_ = 1.0 and *µ*_s_ = 2.0 relative to *µ*_s_ = 0.5 (the light blue plots), as shown in Fig. 5d. In the elderly faller model, the gradual decrease in minimum MOS before fall under high-friction conditions could be attributed to a small stride length and low foot clearance. Early in the stance phase, a small stride length produces small moment around the whole-body CoM in the backward tilt direction^18^. The small foot clearance helps increase the backward horizontal ground reaction force due to increased RCOF^12^, which results in a large moment around the body CoM in the forward tilt direction. Thus, the gait of the elderly faller model gradually becomes unstable in the forward direction, which makes the foot in the swing phase more likely to contact the ground and result in a large ground reaction force that induces a fall.

This study has some limitations that should be mentioned. In our simulation, the heights and weights of the gait models were all the same despite this being inconsistent with statistical data^31,32^. An actual young adult can reduce the probability of falls by taking compensating actions such as adjusting the ankle^33^. However, our gait models did not include a feedback system to perform compensating actions when a large disturbance occurs. Thus, our results indicate only a qualitative trend. Additionally, our models only consider motion in the sagittal plane and not mediolateral motion, which can be a problem for the elderly due to postural instability^34^ and the risk of slipping^30^. Also, our results were solely obtained through computer simulations, experimental verification of our observations is necessary.

## Conclusions

In this study, we used a two-dimensional neuromusculoskeletal bipedal model to generate gait models of a young adult, elderly non-faller, and elderly faller and performed computer simulations to investigate the relation between the foot–ground coefficient of friction and fall risk. Under low-friction conditions, the elderly faller model was the most prone to slip-induced falls as indicated by SL_FC_, which resulted in a large RCOF. Under high-friction conditions, the elderly faller model experienced trip-induced falls, which can be explained by an increasing forward instability increasing the likelihood of the foot in the swing phase contacting the ground, which would induce a large backward ground reaction force and cause a fall. These results indicate there may be an optimal range for the foot–ground coefficient of friction to prevent slip- or trip-induced falls, which provides new insight into the design of shoes and floor surfaces for the elderly. The upper limit of the coefficient of friction should be clarified experimentally in the future.

## Methods

### Neuromusculoskeletal model

We used a two-dimensional neuromusculoskeletal bipedal gait model developed by Taga^24^. Within this model, the gait emerges through self-organization resulting from interactions among three dynamic systems: the musculoskeletal system, neural rhythm generators, and environment. Similar models have been used in studies on gait simulation^35–37^ and robot control theories^38,39^. This model is beneficial for studying the effect of foot–ground friction on fall risks because it automatically adjusts the gait to changes in the environment and falls when the adjustments are not sufficient. Furthermore, the model parameters can be tuned to simulate the effects of the aging process on gait patterns and test fall risks. This allowed us to avoid performing experiments with numerous ground friction conditions and subjecting frail individuals to fall risks.

The model represents the musculoskeletal system as eight rigid segments: the head–arms–trunk (HAT), pelvis, and the right and left thighs, shanks, and feet. The height and mass of the model were set to 1.8 m and 70 kg, respectively. The equations of motion for the segments can be expressed as follows:

1 $$\varvec{M\ddot{x}} = \varvec{A}_{{\text{x}}} \varvec{F}_{{\text{x}}} + \varvec{A}_{{{\text{gx}}}} \varvec{F}_{{{\text{gx}}}}$$

2 $$\varvec{M\ddot{y}} = \varvec{A}_{{\text{y}}} \varvec{F}_{{\text{y}}} + \varvec{A}_{{{\text{gy}}}} \varvec{F}_{{{\text{gy}}}} + \varvec{G}$$

3 $$\varvec{I\ddot{\theta }} = \varvec{B}\left( \theta \right)\varvec{F} + \varvec{B}_{{\text{g}}} \left( \theta \right)\varvec{F}_{{\text{g}}} + \varvec{C}_{{\text{p}}} \varvec{T}_{{\text{p}}} \left( {\theta , \dot{\theta }} \right) + \varvec{C}_{{\text{a}}} \varvec{T}_{{\text{a}}}$$

where ${\varvec{x}}$ and ${\varvec{y}}$ are 8 × 1 vectors of the horizontal and vertical positions, respectively, of the eight segments, and $\varvec{\theta}$ is the inertial angles of the eight segments. ${{\varvec{F}}_{\text{x}}}$ and ${{\varvec{F}}_{\text{y}}}$ are 7 × 1 vectors of the constraint forces in the horizontal and vertical directions, respectively. ${{\varvec{F}}_{{\text{gx}}}}$ and ${{\varvec{F}}_{{\text{gy}}}}$ are 4 × 1 vectors of the ground reaction forces in the horizontal and vertical directions, respectively (${\varvec{F}}=~{\left( {{{\varvec{F}}_{\text{x}}}^{{\text{t}}},~{{\varvec{F}}_{\text{y}}}^{{\text{t}}}} \right)^{\text{t}}}=~{\left( {{{\varvec{F}}_{{\text{gx}}}}^{{\text{t}}},~{{\varvec{F}}_{{\text{gy}}}}^{{\text{t}}}} \right)^{\text{t}}}$). ${{\varvec{T}}_{\text{p}}}$ and ${{\varvec{T}}_{\text{a}}}$ are 7 × 1 vectors of the passive torque exerted by joints and active torque exerted by muscles, respectively. ${\varvec{M}}$ and ${\varvec{I}}$ are 8 × 8 diagonal matrices of the mass and moment of inertia, respectively. ${{\varvec{A}}_{\text{x}}}$ and ${{\varvec{A}}_{\text{y}}}$ are 8 × 7 matrices with constant values. ${{\varvec{A}}_{{\text{gx}}}}$, ${{\varvec{A}}_{{\text{gy}}}}$ are 8 × 4 matrices with constant values. ${\varvec{G}}$ is an 8 × 1 vector of the gravitational force. ${\varvec{B}}\left( \theta \right)$ is an 8 × 14 matrix, ${{\varvec{B}}_{\text{g}}}\left( \theta \right)$ is an 8 × 8 matrix, and ${{\varvec{C}}_{\text{p}}}$ and ${{\varvec{C}}_{\text{a}}}~$are 8 × 7 matrices.

The seven joints of the model (i.e., trunk and the right and left hips, knees, and ankles) each contain a neural oscillator that models the rhythm generation mechanism of the neural system. Each neural oscillator consists of two neurons that activate the flexor and extensor muscles. The outputs $f\left( {{u_{\text{i}}}} \right)$ and $f\left( {{v_{\text{i}}}} \right)$ from the *i*th neuron are represented by the following differential equations:

4 $${\tau _{\text{i}}}{\dot {u}_{\text{i}}}={\text{~}} - {u_{\text{i}}} - {\text{~}}\beta f\left( {{v_{\text{i}}}} \right)+{\text{~}}\mathop \sum \limits_{{j=1}}^{{14}} {w_{{\text{ij}}}}^{0}f\left( {{u_{\text{i}}}} \right)+{u_0}+{\text{~}}{Q_{\text{i}}}+{\text{~}}{S_{\text{i}}}$$

5 $${\dot {\tau }_{\text{i}}}{\dot {v}_{\text{i}}}=~ - {v_{\text{i}}}+~f\left( {{u_{\text{i}}}} \right)$$

6 $$f\left( u \right)=\hbox{max} \left( {0,u} \right)$$

where *i* (= 1–14) represents a given neuron. ${u_{\text{i}}}$ is the inner state of the *i*th neuron. ${v_{\text{i}}}$ is a variable that represents the degree of the adaptation or self-inhibition effect of the *i*th neuron. ${\tau _{\text{i}}}$ and ${\dot {\tau }_{\text{i}}}$ are the time constants of the inner state and adaptation effect, respectively, of the *i*th neuron. $\beta$ is a coefficient of the adaptation effect. ${w_{{\text{ij}}}}$ is a connecting weight from the *j*th neuron to the *i*th neuron. ${u_0}$ is the nonspecific steady input that determines the active state of the neuron. ${Q_{\text{i}}}$ is the input signal sent from other neurons to the *i*th neuron. ${S_{\text{i}}}$ is the feedback sent to the *i*th neuron.

**Fig. 6:**
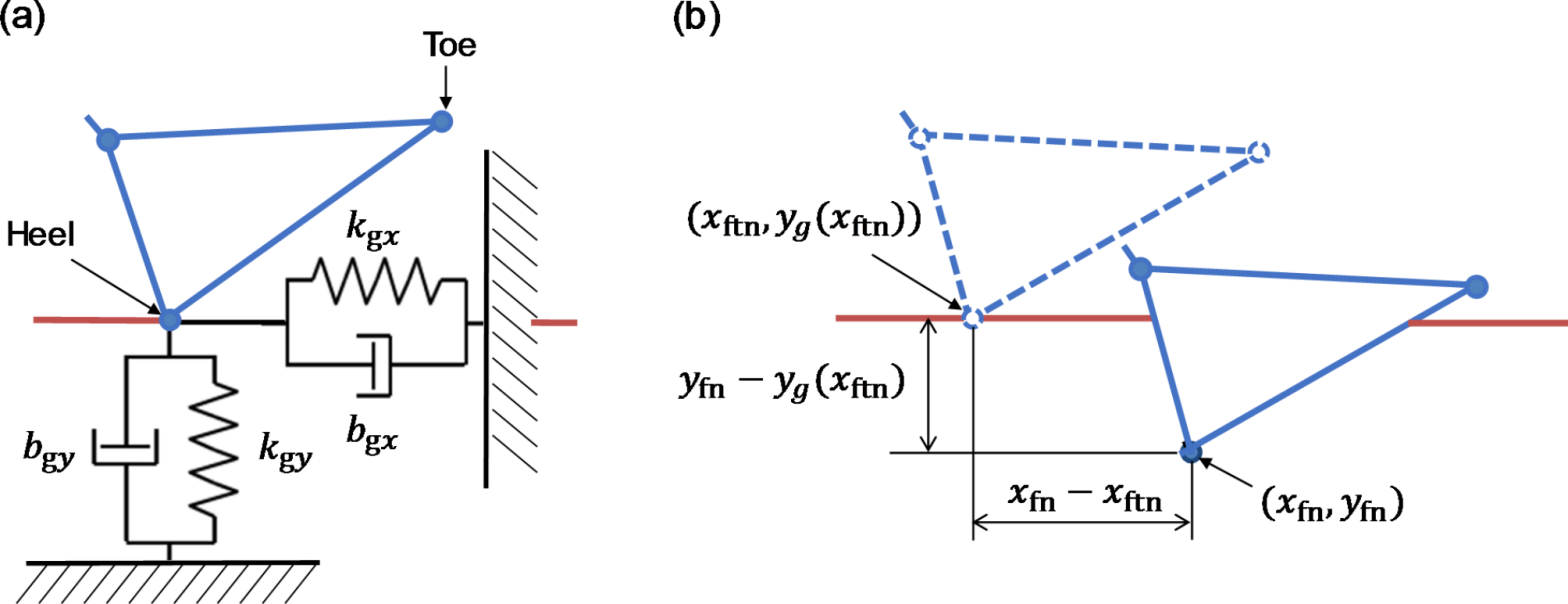
Schematic diagram of contact between the foot and ground: (a) representation as spring–damper systems in the horizontal and vertical directions and (b) displacement of the ground surface in the *x* and *y* directions when contacted by the heel. ${k_{{\text{gx}}}}$ = 27,000 N/m, ${k_{{\text{gy}}}}$ = 18,000 N/m, ${b_{{\text{gx}}}}$ = 2250 N s/m, and ${b_{{\text{gy}}}}$ = 1000 N s/m.

The model contacts the ground at four points: the right and left toes and heels. The contact at each point can be represented as spring–damper systems in the horizontal and vertical directions (Fig. 6a). The ground reaction forces in the horizontal direction (${F_{{\text{gxn}}}}$) and vertical direction (${F_{{\text{gyn}}}}$) acting on a contact point (${x_{{\text{fn}}}}$, ${y_{{\text{fn}}}}$) are obtained as follows: (Fig. 6b):

7 $${F_{{\text{gxn}}}}=\left\{ { - {k_{{\text{gx}}}}\left( {{x_{{\text{fn}}}} - {\text{~}}{x_{{\text{ftn}}}}} \right) - {\text{~}}{b_{{\text{gx}}}}{x_{{\text{fn}}}}} \right\}{\text{~}}1\left( {{y_{\text{g}}}({x_{{\text{ftn}}}}) - {y_{{\text{fn}}}}} \right)$$

8 $$F_{{{\text{gyn}}}} = \left\{ { - k_{{{\text{gy}}}} \left( {y_{{{\text{fn}}}} - ~y_{{\text{g}}} \left( {x_{{{\text{ftn}}}} } \right)} \right) + ~b_{{{\text{gy}}}} f\left( { - \mathop {y_{{{\text{fn}}}} }\limits^{.} } \right)} \right\}~1\left( {y_{{\text{g}}} (x_{{{\text{ftn}}}} ) - y_{{{\text{fn}}}} } \right)$$

9 $$1\left( x \right)=~\left\{ {\begin{array}{*{20}{c}} {1~\left( {0.01<x} \right)} \\ {100x~\left( {0 \leqslant x \leqslant 0.01} \right),~f\left( x \right)=\hbox{max} \left( {0,x} \right)} \\ {0~\left( {x<0} \right)} \end{array}} \right.$$

where *n* (1–4) represents a given contact point (i.e., right and left heels and toes). The coordinates (${x_{{\text{ftn}}}}$, ${y_{\text{g}}}({x_{{\text{ftn}}}})$) indicate the rest position of the elastic forces, which are reset each time the heels and/or toes contact the ground. ${y_{\text{g}}}\left( x \right)$ is a function of the ground profile that is established along the sagittal plane such that the elevation of the terrain changes horizontally. ${k_{{\text{gx}}}}$ and ${k_{{\text{gy}}}}$ are spring coefficients in the horizontal and vertical directions, respectively, and ${b_{{\text{gx}}}}$ and ${b_{{\text{gy}}}}$ are damper coefficients in the horizontal and vertical directions, respectively. In this study, the spring and damper coefficients of the ground were set as constant (${k_{{\text{gx}}}}$ = 27,000 N/m, ${k_{{\text{gy}}}}$ = 18,000 N/m, ${b_{{\text{gx}}}}$ = 2250 N s/m, and ${b_{{\text{gy}}}}$ = 1000 N s/m). This model allows the gait as well as reactive movements to mechanical perturbations emerge in a self-organized manner without preset joint kinematics^24^.

### Gait models

We created three gait models simulating a young adult, elderly non-faller, and elderly faller. The young adult model was the original model used in the literature^24^. The elderly non-faller and faller models were created by changing three parameters of the neuromusculoskeletal model (Table 2): the ratio ${\tau _{\text{i}}}$/${\dot {\tau }_{\text{i}}}$, transfer coefficients *p*_k_ (*k* = 1–18 corresponding to the number of joint torque inputs), and nonspecific steady input ${u_0}$. ${\tau _{\text{i}}}/{\dot {\tau }_{\text{i}}}$ was set to achieve less fluctuating gaits for the young adult model and elderly non-faller model and a more fluctuating gait for the elderly faller model. *p*_k_ is the coefficient for the magnitude of the contribution from a neural oscillator to the torque of a particular muscle system, and the magnitude of joint torque (extension and flexion) was adjusted at each lower limb joint^24^. The torque was also adjusted by changing the value of the input from the neural oscillator by changing *u*_0_^24^. In the elderly non-faller model, the *u*_0_ was lower than that in the younger adult model, and for the elderly faller model, the joint torque was decreased by decreasing *u*_0_ and *p*_k_ relative to the elderly non-faller model. These parameters were set to reproduce differences in the gait parameters for each age group (healthy young adult, elderly non-faller, and elderly faller groups) obtained from the gait experiments in previous literature^25–28^. Adding white noise to ${u_0}$ (as a percentage of ${u_0}$) induced more natural fluctuations in the gait^40^.

**Table 2:** Values of ${\tau _{\text{i}}}$/${\dot {\tau }_{\text{i}}}$, *p*_k_, and ${u_0}$ used for the three gait models.

	Young adult model	Elderly non-faller model	Elderly faller model
*u*_0_	7.08	5.16	5.10
${\tau _i}/{\dot {\tau }_i}$	0.036	0.100	0.076
*p*_1_	5.00	5.00	4.75
*p*_2_	10.00	10.00	9.50
*p*_3_	4.00	4.00	2.66
*p*_4_	2.00	2.00	0.86
*p*_5_	15.00	15.00	12.83
*p*_*6*_	4.00	4.00	3.42
*p*_7_	3.00	3.00	2.85
*p*_8_	2.00	2.00	1.90
*p*_9_	15.00	15.00	14.25
*p*_10_	8.00	8.00	7.60
*p*_11_	2.00	2.00	1.90
*p*_12_	3.00	3.00	2.85
*p*_13_	2.00	2.00	1.90
*p*_14_	8.00	8.00	3.92
*p*_15_	1.50	1.50	0.90
*p*_16_	12.00	12.00	10.26
*p*_17_	1.00	1.00	0.86
*p*_18_	7.00	7.00	5.99
Noise, %	8.50	2.00	0.100

Notes: ${\tau _{\text{i}}}$/${\dot {\tau }_{\text{i}}}$: ratio of time constants; *p*_k_: transfer coefficients; ${u_0}$: nonspecific steady input to which white noise was added. *i* (1–14) indicates a given neuron, and *k* (1–18) indicates a given muscle torque input.

Slips theoretically occur when the ratio of the horizontal force to the vertical force applied onto the ground (i.e., traction coefficient ${F_{{\text{gxn}}}}/{F_{{\text{gyn}}}}$) by the foot reaches the static coefficient of friction *µ*_s_. When${F_{{\text{gxn}}}}/{F_{{\text{gyn}}}}$ < *µ*_s_ throughout the stance phase, slips do not occur. In this study, we set *µ*_s_ between the foot and ground and the horizontal ground reaction force ${F_{{\text{gxn}}}}$ as follows depending on the relation between ${F_{{\text{gxn}}}}/{F_{{\text{gyn}}}}$ and ${\mu _{\text{s}}}$:

10 $$\begin{array}{l}{F_{{\text{gxn}}}}/{F_{{\text{gyn}}}}{\text{~}} \leqslant {\text{~}}{\mu _{\text{s}}}{\text{~}}:{F_{{\text{gxn}}}}={F_{{\text{gxn}}}} \\{F_{{\text{gxn}}}}/{F_{{\text{gyn}}}}{\text{~}}>{\text{~}}{\mu _{\text{s}}}{\text{~}}:{F_{{\text{gxn}}}}={\mu _{\text{s}}}{F_{{\text{gyn}}}}\end{array}$$

${F_{{\text{gxn}}}}/{F_{{\text{gyn}}}}$ can take a larger value at a higher *µ*_s_ than at a lower *µ*_s_. We set *µ*_s_ to values in the range of 0.050–2.0, which corresponds to real-world conditions such as icy surfaces^41,42^, wet surfaces^43^, and dry surfaces^44–46^. Under conditions of high friction, the maximum value was set to 2.0 because the coefficient of friction between the rubber and the smooth floor surface is greater than 1.5^45,46^. *μ*_s_ was varied in increments of 0.050 over the range 0.050 ≤ *μ*_s_ ≤ 0.50, and in 0.1 increments over the range 0.50 < *μ*_s_ ≤ 2.0. Five trials were performed with each gait model under each *µ*_s_ condition. White noise was applied to induce fluctuations in the gait with each step even for the same gait model. *µ*_s_ was changed after 5 m when the gait parameters became stable. Before the initial 5 m, the value of *µ*_s_ was not set, and ${F_{{\text{gxn}}}}/{F_{{\text{gyn}}}}$ could take any value.

A fall was defined as when the hip height fell below 0.70 m^47^ within 10 m after *µ*_s_ was changed (i.e., 5–15 m from the start). A slip was defined as when a foot in the stance phase moved at a velocity greater than 0.3 m/s^12,48,49^. A trip-induced fall was identified when a fall occurred in the forward direction with the foot in the stance phase moving at a velocity of less than 0.3 m/s. Based on the simulation results, we examined the effect of *µ*_s_ on falls by the three gait models. We extracted the maximum value of ${F_{{\text{gxn}}}}/{F_{{\text{gyn}}}}$ ((*F*_gxn_/*F*_gyn_)_max_) and investigated whether it reached *µ*_s_ in each trial.

### Data analysis

To validate the differences between the gait models, the mean and CV values of stride length, walking speed, and minimum and maximum foot clearance were calculated for 10 consecutive strides of a stable gait for each model. The stride length was the stride-to-stride distance measured at the right toe. The minimum foot clearance was calculated as the minimum vertical distance between the toe and the floor during the mid-swing phase, and the maximum foot clearance was calculated as the maximum vertical distance between the heel and the floor during the mid-swing phase. The gait speed was calculated by dividing the stride length by the time between strides, and the cadence was the reciprocal of the stride-to-stride time.

To investigate the relationship between the age-related changes in stride length and foot clearance in each model and a slip risk, the relationship between the value of stride length/foot clearance (SL_FC_) and the maximum static friction coefficient at which slip-induced falls occur (*µ*_s-slip_).

The MOS, an index of gait stability, was calculated to evaluate the effect of *µ*_s_ on postural stability in each gait model according to the following equation^50^:

11 $$MOS=BOS - XCoM,~{\text{where~}}XCoM=X+~\frac{V}{{\sqrt {{\raise0.7ex\hbox{$g$} \!\mathord{\left/ {\vphantom {g l}}\right.\kern-0pt}\!\lower0.7ex\hbox{$l$}}} }}$$

where $BOS$ is the boundary of the base of support and $XCoM$ is the location of the extrapolated CoM. $BOS$ was defined by the front and back of foot (anterior and posterior borders). The anterior boundary is the toe of the leading foot during the double-support phase and toe of the standing foot during the single-stance phase. The posterior boundary is the heel of the trailing foot during the double-support phase and the heel of standing foot during the single-stance phase. *X* is the position of the body CoM, and *V* is the velocity of the body CoM. *g* is the gravitational constant, and *l* is the effective pendulum length. Because a forward slip causes backward imbalance and a trip causes forward imbalance, the MOS was calculated for the posterior BOS border (the heel) and the anterior BOS border (the toe), as shown in Fig. 5a. The MOS equation was adjusted to account for the direction of the coordinate system, so that positive and negative MOS values indicated stable (XCoM within BOS) and unstable (XCoM outside BOS) states, respectively^51^. Therefore, when the XCoM was in front of the anterior BOS boundary (the toe) in the direction of travel, the anterior MOS was negative (unstable), and when the XCoM is behind the posterior BOS boundary (the heel) in the direction of travel, the MOS was also negative (unstable). The minimum value of the anterior and posterior MOS in each step was used, and the change in the minimum MOS values with respect to number of steps after a change of *µ*_s_ was evaluated for each gait model. All the simulations and analyses were performed using MATLAB (Ver. R2023a, MathWorks, Natick, MA, USA).

## Electronic supplementary material

Below is the link to the electronic supplementary material.

Supplementary Material 1

Supplementary Material 2

Supplementary Material 3

Supplementary Material 4

Supplementary Material 5

Supplementary Material 6

Supplementary Material 7

## Data Availability

The data that support the findings of this study are available from the corresponding author upon reasonable request.
