# Supplementary material for: Effects of foot–ground friction and age-related gait changes on falls during walking: a computational study using a neuromusculoskeletal model
Source: Sci Rep. 2024 Nov 28;14:29617. doi: 10.1038/s41598-024-81361-7 (PMC11605121; doi:10.1038/s41598-024-81361-7)
Supplement: Supplementary file 7 — Supplementary Material 7 [file 41598_2024_81361_MOESM7_ESM.docx]

**Fig. A1.** Relation between the maximum absolute value of $F_{\mathrm{gxn}}/F_{\mathrm{gyn}}$ in each trial ((*F*_gxn_/*F*_gyn_)_max_) and falls: (a) the young adult model, (b) elderly non-faller model, and (c) elderly faller model. The black dots represent no-falls (0), and the crosses represent falls (1). Blue falls are slip-induced, and red falls are trip-induced. Each graph includes all simulation results and thus has 125 plots.
